# Loss of the p53/p63 Regulated Desmosomal Protein Perp Promotes Tumorigenesis

**DOI:** 10.1371/journal.pgen.1001168

**Published:** 2010-10-21

**Authors:** Veronica G. Beaudry, Dadi Jiang, Rachel L. Dusek, Eunice J. Park, Stevan Knezevich, Katie Ridd, Hannes Vogel, Boris C. Bastian, Laura D. Attardi

**Affiliations:** 1 Department of Radiation Oncology, Division of Radiation and Cancer Biology, Stanford University School of Medicine, Stanford, California, United States of America; 2 Department of Pathology, Stanford University School of Medicine, Stanford, California, United States of America; 3 Department of Dermatology, University of California San Francisco, San Francisco, California, United States of America; 4 Department of Pathology and UCSF Helen Diller Family Comprehensive Cancer Center, University of California San Francisco, San Francisco, California, United States of America; 5 Department of Genetics, Stanford University School of Medicine, Stanford, California, United States of America; Fred Hutchinson Cancer Research Center, United States of America

## Abstract

Dysregulated cell–cell adhesion plays a critical role in epithelial cancer development. Studies of human and mouse cancers have indicated that loss of adhesion complexes known as adherens junctions contributes to tumor progression and metastasis. In contrast, little is known regarding the role of the related cell–cell adhesion junction, the desmosome, during cancer development. Studies analyzing expression of desmosome components during human cancer progression have yielded conflicting results, and therefore genetic studies using knockout mice to examine the functional consequence of desmosome inactivation for tumorigenesis are essential for elucidating the role of desmosomes in cancer development. Here, we investigate the consequences of desmosome loss for carcinogenesis by analyzing conditional knockout mice lacking *Perp*, a p53/p63 regulated gene that encodes an important component of desmosomes. Analysis of *Perp*-deficient mice in a UVB-induced squamous cell skin carcinoma model reveals that *Perp* ablation promotes both tumor initiation and progression. Tumor development is associated with inactivation of both of Perp's known functions, in apoptosis and cell–cell adhesion. Interestingly, *Perp*-deficient tumors exhibit widespread downregulation of desmosomal constituents while adherens junctions remain intact, suggesting that desmosome loss is a specific event important for tumorigenesis rather than a reflection of a general change in differentiation status. Similarly, human squamous cell carcinomas display loss of PERP expression with retention of adherens junctions components, indicating that this is a relevant stage of human cancer development. Using gene expression profiling, we show further that *Perp* loss induces a set of inflammation-related genes that could stimulate tumorigenesis. Together, these studies suggest that *Perp*-deficiency promotes cancer by enhancing cell survival, desmosome loss, and inflammation, and they highlight a fundamental role for Perp and desmosomes in tumor suppression. An understanding of the factors affecting cancer progression is important for ultimately improving the diagnosis, prognostication, and treatment of cancer.

## Author Summary

Changes in tissue architecture, such as loss of adhesion between cells, have been shown to facilitate cancer development, especially metastasis where cells can detach from a tumor and spread throughout the body. While various studies have demonstrated that inactivation of an adhesion complex known as the adherens junction promotes cancer development and metastasis, little is known about the role of the desmosome—a related cell–cell adhesion complex—in tumorigenesis. Here we examine the consequence of desmosome-deficiency for tumor development by studying mice lacking a key component of desmosomes in the skin, a protein known as Perp. Using a mouse model for human skin cancer, in which ultraviolet light promotes skin cancer development, we demonstrate that Perp-deficiency indeed leads to accelerated skin tumorigenesis. We similarly observe that PERP is lost during human skin cancer development, suggesting that PERP is also important as a tumor suppressor in humans. These findings demonstrate that desmosome-deficiency achieved by Perp inactivation can promote cancer and suggest the potential utility of monitoring PERP status for staging, prognostication, or treatment of human cancers.

## Introduction

Carcinomas, or cancers of epithelia, comprise approximately 90% of all human cancers [1]. Cancers of the stratified epithelia, such as the skin and the tissues of the head and neck, are common and can display poor prognosis in advanced stages [2], [3]. Elaborating the normal architecture and homeostatic mechanisms of stratified epithelia, and how these are perturbed in cancer progression and metastasis, can provide significant insight into carcinoma development.

The genesis and maintenance of stratified epithelia, such as the epidermis, requires the coordinated regulation of proliferation, adhesion, migration, and differentiation [4]. Progenitor cells of the basal, inner layer of the epidermis renew the epidermis by initially proliferating, then exiting the cell cycle, detaching from the basement membrane, migrating, and differentiating to form the upper layers of the skin. The cells of stratified epithelia require a great deal of plasticity to permit division and migration of cells during the differentiation process while still providing a protective barrier to prevent dehydration and infection. This plasticity relies on the modulation of various intercellular adhesion junctions, including tight junctions, adherens junctions and desmosomes [5]. While adherens junctions generally help to maintain cell-cell adhesion in epithelial tissues, desmosomes have been found to be particularly important for endowing epithelia with the strength needed to withstand mechanical stress, via anchorage to the intermediate filament network [5], [6]. The crucial nature of desmosomes in maintaining the integrity of stratified epithelia is underscored by the observation that mutation or inactivation of several desmosomal proteins is linked to human epithelial diseases [7].

The importance of dysregulated adhesion in cancer has been best understood from analysis of adherens junctions. Adherens junctions promote intercellular adhesion through homotypic interactions between transmembrane E-cadherin molecules at the plasma membrane [8], [9] and connections to the actin cytoskeleton via alpha-catenin and beta-catenin [10], [11]. In human cancers, inactivation of E-cadherin, through mutation, promoter methylation, or transcriptional repression, is associated with epithelial to mesenchymal transition (EMT), progression from adenoma to carcinoma, and the acquisition of metastatic potential [12]–[16]. The importance of this inactivation is underscored by functional studies in mouse models showing that inhibition of E-cadherin in certain tumor-prone strains facilitates invasion and metastasis [17]. In addition, compound mutant mice lacking both *E-cadherin* and *p53* in the mammary epithelium exhibit accelerated tumor development and increased metastasis relative to mice lacking only *p53* [18]. Loss of p120-catenin, a key regulator of E-cadherin stability, also promotes neoplasia, in the salivary gland and the skin [19]–[21]. Similarly, specific deletion of *Alpha-catenin* in the mouse epidermis results in squamous cell carcinoma development [21], [22].

While a variety of studies have implicated inactivation of adherens junctions in tumor development and progression, the contribution of desmosome loss to carcinogenesis remains largely unexplored. Desmosome complexes form when the desmosomal cadherins, desmogleins and desmocollins, participate in heterotypic interactions that bring the plasma membranes of adjacent cells in close apposition [23]–[25]. The cytoplasmic tails of these cadherins interact with plakoglobin [26], [27] and plakophilins [28]–[30], which connect to the intermediate filament cytoskeleton via desmoplakin [31]–[33]. An impediment to studying desmosomes in a genetic cancer model has been the high frequency of embryonic or perinatal lethality observed in various knockout mice lacking desmosomal components, precluding long-term tumor studies [34]. Additionally, correlative studies examining expression patterns of desmosomal components during human cancer progression have yielded conflicting results. Several studies have suggested that downregulation of desmosome components, including desmoglein 3, desmoglein 2, plakoglobin, and desmoplakin, occurs during the progression of a variety of cancers in humans and is often correlated with and predictive of tumor metastasis [35]–[38]. In contrast, other studies have documented the overexpression of desmosome components during the progression of diverse cancers, and this pattern is associated with poor prognosis [39]–[41]. The use of tractable genetic systems is therefore critical for unraveling the contribution of desmosomes to cancer development.

The Perp tetraspan membrane protein was originally identified as a transcriptional target of the p53 tumor suppressor upregulated during apoptosis [42]. Subsequent analysis of *Perp* knockout mice revealed an additional function for Perp as a target of the p53-related transcription factor, p63, involved in maintaining epithelial integrity by promoting desmosomal cell-cell adhesion [43]. *Perp*−/− mice display postnatal lethality accompanied by dramatic blisters throughout their stratified epithelia, including the oral mucosa and skin. Electron microscopy and biochemical analyses established that the blistering phenotype observed in the *Perp*-deficient epithelia is accounted for by both a reduction in desmosome number and compromised desmosome complex formation. Immunogold electron microscopy demonstrating localization of Perp to desmosomes conclusively established a clear role for Perp as a novel critical component of the desmosome in the skin and other stratified epithelia.

Here, we aim to characterize the consequence of *Perp*-deficiency for UVB-induced squamous cell carcinoma (SCC) development in the skin, where Perp plays a pivotal role in cell-cell adhesion and tissue homeostasis. Using conditional *Perp* knockout mice to selectively ablate *Perp* expression in stratified epithelia, we reveal an important role for Perp as a tumor suppressor in this model for human skin cancer. These results provide definitive genetic evidence that loss of a desmosome component can in fact promote tumorigenesis *in vivo*, thereby enhancing our understanding of the factors driving tumor progression.

## Results

### Loss of Perp promotes tumorigenesis

**Figure 1:**
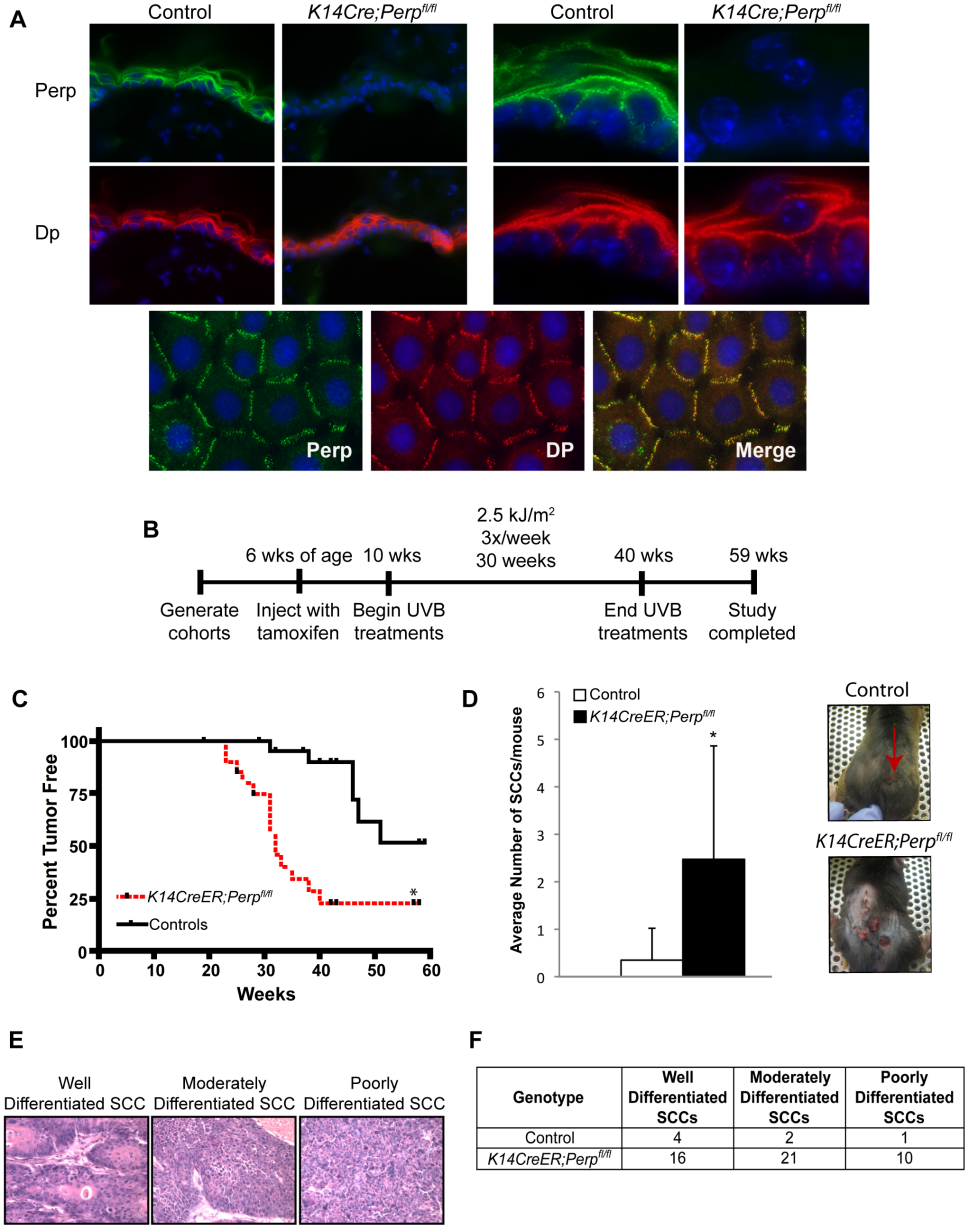
*Perp*-deficiency promotes tumorigenesis. A) Perp immunofluorescence images demonstrating the presence of Perp in the epidermis of control mice and the loss of Perp in the epidermis of *K14CreER;Perp^fl/fl^* mice. Green signal represents Perp staining, red signal represents staining for the desmosomal protein Desmoplakin (Dp), and blue signal represents DAPI, to mark nuclei. (Upper left) Low magnification images show an overview of the epidermis, and (Upper right) high magnification images show the punctate staining pattern typical of desmosomal proteins. (Lower panel) Punctate desmosomal pattern in the epidermis is comparable to that observed in wild-type mouse keratinocyte monolayers. B) Tumor study design. C) Kaplan-Meier analysis showing tumor latency in UVB-treated control (*Perp^fl/fl^* and *Perp^fl/+^*) and *K14CreER;Perp^fl/fl^* mice. Statistical significance was determined using the Log Rank test (* p = 0.0002). n =  25 for each genotype. D) (Left) Graph depicts the average number of SCCs per UVB-treated mouse +/− STDEV. Statistical analysis was performed using the Student's unpaired t-test (* p =  0.00049). (Right) Representative photographs of tumor burden in control and *K14CreER;Perp^fl/fl^* mice, with arrow indicating a tumor. E) Representative Hematoxylin and Eosin (H&E) stained images illustrating the various SCC grades. F) Table indicating the numbers of SCCs of different grades in UVB-treated control and *K14CreER;Perp^fl/fl^* mice.

To characterize the function of Perp during tumorigenesis, we examined its role in cancer of the epidermis, where it is critical for tissue integrity and homeostasis through its role in desmosomal cell-cell adhesion [43]. We examined the tumor predisposition of *Perp*-deficient mice using a well-defined model for squamous cell carcinoma (SCC) development in which mice are exposed to chronic UVB irradiation [44]. This model provides an accurate mimic of human SCC development, which is similarly driven by UVB irradiation. As *Perp* constitutive null mice die postnatally, we utilized conditional *Perp* knockout mice (*Perp^fl/fl^*; fl = floxed) expressing a tamoxifen-inducible *K14CreER* transgene to drive tissue-specific deletion of the *Perp* locus in the epidermis [45], [46]. Immunofluorescence confirmed that Perp expression was successfully ablated in the epidermis of the majority of these mice 4 weeks after tamoxifen injection (Figure 1A). To induce SCC development, tamoxifen-treated 10-week old control and *Perp^fl/fl^* mice expressing a *K14CreER* transgene were exposed to chronic treatments (2.5 kJ/m^2^) of UVB irradiation three times weekly for 30 weeks (Figure 1B). Interestingly, Kaplan-Meier analysis revealed that mice lacking Perp in the epidermis developed SCCs with reduced average latency (32 wks) compared to control mice (51 wks; Figure 1C). In addition, the average number of SCCs per *K14CreER;Perp^fl/fl^* mouse was far greater than in control animals (Figure 1D). The prominent early tumor development and increased tumor number in Perp-deficient mice compared to controls suggest that Perp loss promotes tumor initiation. Histological analyses to grade the SCCs according to cellular morphology, invasiveness into the dermis, and overall architecture revealed that SCCs arising in *K14CreER;Perp^fl/fl^* mice had a greater propensity to progress to a poorly differentiated stage than tumors arising in control mice, suggesting that Perp loss may also contribute to tumor progression (Figure 1E, 1F). Despite the presence of invasive tumors, however, no metastases were apparent in the liver or lungs of mice from either cohort (data not shown). Together, these findings indicate that Perp is a key suppressor of skin carcinogenesis and provide the first *in vivo* demonstration that genetic loss of a desmosomal component can lead to accelerated carcinoma development, facilitating both tumor initiation and progression.

### Perp is an important mediator of UVB-induced apoptosis

**Figure 2:**
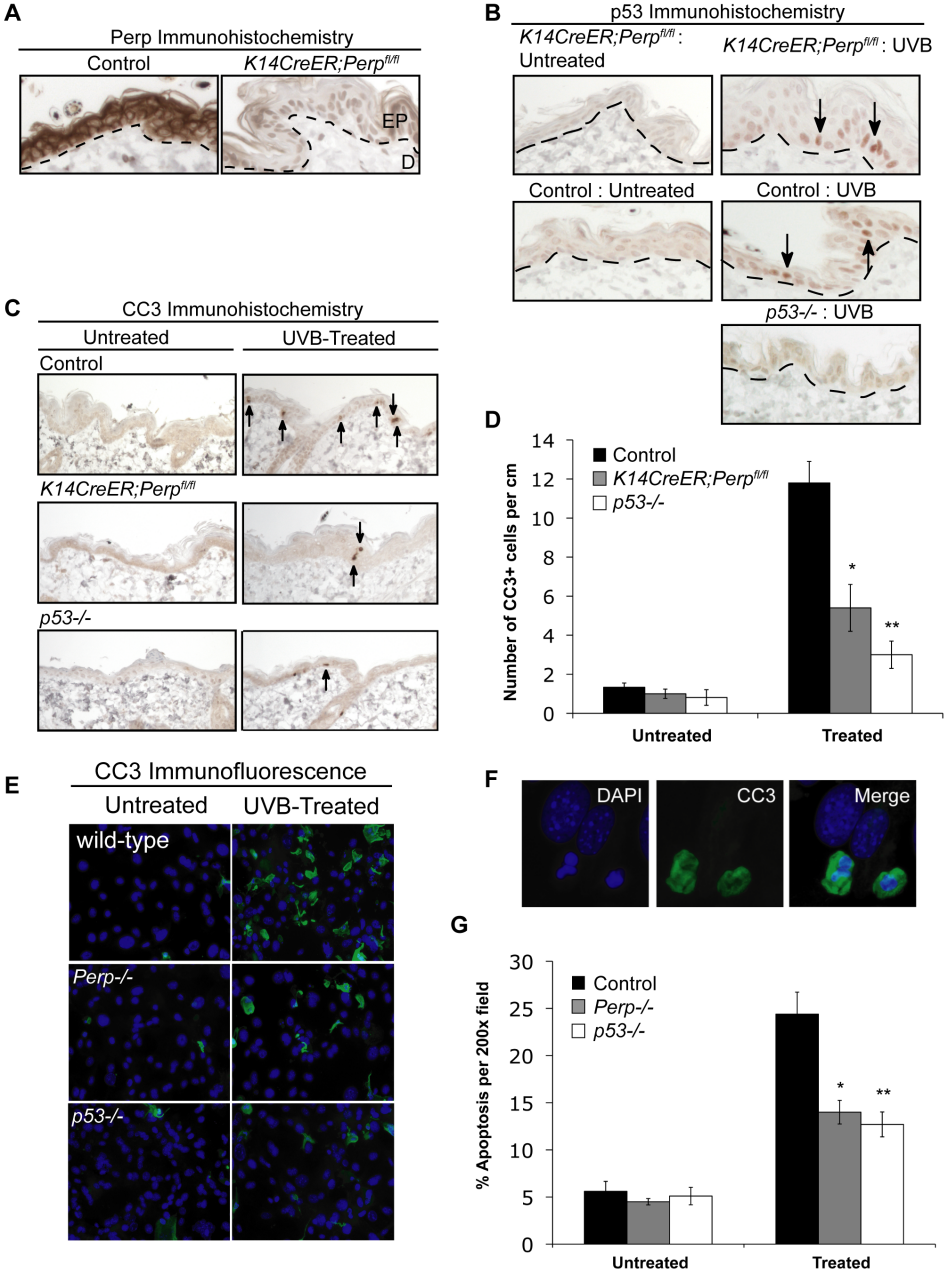
Perp loss compromises UVB-induced apoptosis *in vivo* and *in vitro*. A) Perp immunohistochemistry showing loss of Perp in the epidermis of tamoxifen-treated *K14CreER;Perp^fl/fl^* mice. Dashed line demarcates epidermis (EP) and dermis (D). B) Immunohistochemistry showing p53 stabilization (arrows) in the epidermis of both control and *K14CreER;Perp^fl/fl^* mice 24 hrs after treatment with 2.5 kJ/m^2^ UVB radiation. C) Cleaved Caspase 3 (CC3) immunohistochemistry to detect apoptosis in the epidermis of untreated and UVB-treated mice of different genotypes. Arrows indicate apoptotic cells. D) Quantification of apoptosis in untreated and UVB-treated control, *K14CreER;Perp^fl/fl^*, and *p53−/−* mice. Graph depicts the average number of cleaved Caspase 3 (CC3)- positive cells per linear cm of epidermis, +/− SEM. Data were derived from the analysis of segments of skin at least 2–3 cm long per mouse, in several independent experiments with the following numbers of mice: wild-type controls (n = 8), *K14CreER;Perp^fl/fl^* (n = 12), *p53−/−* (n = 5). Statistical analysis was conducted using the Student's unpaired t-test (* p = 0.0017 versus treated wild-type and ** p = 0.0003 versus treated wild-type). (E) Representative immunofluorescence images of wild-type, *Perp−/−*, and *p53−/−* keratinocyte monolayers, either untreated or treated with 1 kJ/m^2^ UVB, and stained with a cleaved Caspase 3 antibody and DAPI to measure apoptosis. (F) Higher magnification images show apoptotic cells, which display both cleaved Caspase 3-positivity and nuclear blebbing and chromatin condensation by DAPI staining, hallmarks of apoptosis (G) Quantitation of the percentage of apoptotic cells per 200× field in untreated and UVB-treated wild-type, *Perp−/−*, and *p53−/−* keratinocytes, as assessed by cleaved Caspase 3/DAPI staining. Graph represents the average +/− SEM of three independent experiments performed in triplicate. Statistical analysis was conducted using the Student's unpaired t-test. (* p = 0.011 versus treated wild-type and ** p = 0.0029 versus treated wild-type).

To understand the basis for the tumor development driven by *Perp*-deficiency, we sought first to determine whether Perp is an important mediator of p53-induced apoptosis in the skin in response to ultraviolet light. Perp plays a cell-type-specific role in p53-mediated apoptosis, being dispensable for apoptosis in fibroblasts but essential for apoptosis of thymocytes and embryonic neurons in response to DNA damage signals [47]. To examine Perp's role in p53-dependent apoptosis in keratinocytes, 6-week old *K14CreER;Perp^fl/fl^* and control mice were injected with tamoxifen (which ablated Perp expression in *K14CreER;Perp^fl/fl^* mice; Figure 2A), and 4 weeks later, mice were exposed to one dose of 2.5 kJ/m^2^ of UVB radiation. We confirmed that p53 is induced 24 hours after UVB treatment in the epidermis of both wild-type and *K14CreER;Perp^fl/fl^* mice (Figure 2B). Apoptotic indices were then determined by quantifying the number of cleaved Caspase 3-positive cells in the epidermis. Minimal apoptosis was detected in untreated skin of mice of all genotypes (Figure 2C, 2D). While robust apoptosis was observed in the epidermis of wild-type mice in response to UVB radiation, *p53* null mice displayed significantly attenuated levels of apoptosis (Figure 2C, 2D). Interestingly, analysis of the epidermis of *Perp*-deficient mice also revealed diminished apoptosis levels, to an extent nearly equivalent to *p53* loss (Figure 2C, 2D). We confirmed these findings by assaying apoptosis in differentiated keratinocytes *in vitro*, through analysis of both cleaved Caspase 3 positivity and classical apoptotic nuclear morphology and condensed chromatin by DAPI staining [48], [49]. We found that *Perp−/−* keratinocytes displayed defective apoptosis in response to UVB, similar to *p53−/−* keratinocytes (Figure 2E–2G). Together, these results demonstrate that Perp plays an important role in UVB-induced apoptosis in both the skin *in vivo* and keratinocytes *in vitro*. As p53 inactivation is proposed to promote UVB-induced carcinogenesis by allowing inappropriate survival of cells sustaining UVB-induced damage [50], enhanced survival of *Perp*-deficient cells after exposure to ultraviolet light could similarly enable tumor initiation.

### Desmosome components, but not adherens junction components, are selectively downregulated during tumorigenesis

**Figure 3:**
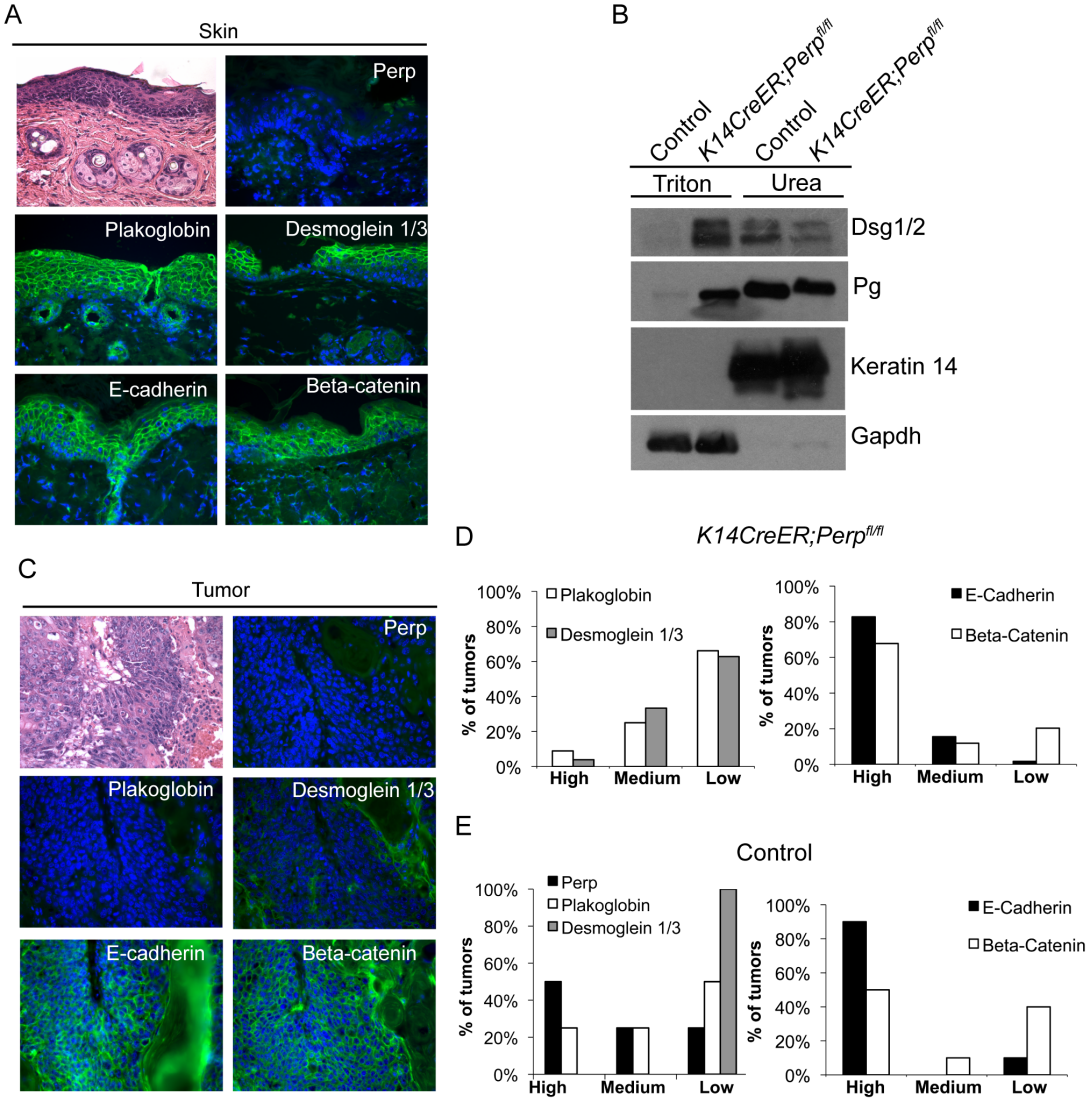
Adhesion junction analysis in tumors from *K14CreER;Perp^fl/fl^* and control mice. A) Representative Hematoxylin and Eosin (H&E) staining and immunofluorescence images of desmosome and adherens junction component protein expression in skin from tamoxifen-treated *K14CreER;Perp^fl/fl^* mice demonstrating that Perp loss itself does not disrupt membrane expression of other adhesion proteins. B) Western blot analysis showing both the Triton X-100-soluble and urea-only soluble fractions of mouse epidermal lysates from control and *K14CreER;Perp^fl/fl^* mice. Desmoglein 1/2 (Dsg 1/2) and Plakoglobin (Pg) solubility were examined. Gapdh serves as a loading control for the Triton X-100-soluble pool, while Keratin 14 serves as the loading control for the urea fraction. C, D) Tumors from *K14CreER;Perp^fl/fl^* mice were stained with antibodies against various desmosomal and adherens junction components, and both the percentage of epithelial cells staining positive for each marker and the intensity of staining were measured. Staining for each antigen was categorized as high (>70%), medium (30–70%), or low (<30%) level expression. C) Representative H&E and immunofluorescence images of a *K14CreER;Perp^fl/fl^* tumor sample demonstrating desmosomal component loss with retention of adherens junction components. Green signal indicates antigen staining, and blue signal indicates DAPI-marked nuclei. D) (Left) Graph showing the percentages of *K14CreER;Perp^fl/fl^* tumors categorized into respective groups based on quantitative immunofluorescence staining for Plakoglobin and Desmoglein 1/3. (Right) Graph depicting the percentages of tumors categorized into respective groups based on immunofluorescence staining for E-cadherin and Beta-catenin. (E) Tumors from control wild-type mice were stained for desmosomal and adherens junction components, and each component was categorized as displaying high, medium, or low level expression, as described above. (Left) Graph showing the percentages of control tumors categorized into respective groups based on quantitative immunofluorescence staining for Perp, Plakoglobin, and Desmoglein 1/3. (Right) Graph depicting the percentages of tumors categorized into respective groups based on immunofluorescence staining for E-cadherin and Beta-catenin.

As a key desmosomal constituent in the epidermis, Perp loss could also promote cancer through effects on cell-cell adhesion. In constitutive *Perp* knockout mice, Perp loss does not abrogate desmosome formation, but leads to decreased numbers of desmosomes and reduced stability of desmosomal components [43]. Simple immunofluorescence analysis of desmosome proteins in *Perp−/−* newborn skin or *K14CreER;Perp^fl/fl^* adult mouse skin does not show perturbed membrane localization of desmosomal components ([43], Figure 1A, Figure 3A), and thus it is necessary to use a biochemical assay to show that desmosomes are functionally compromised upon Perp loss. This solubility assay is based on the fact that properly formed desmosomal complexes can only be solubilized by chaotropic agents, whereas improperly assembled desmosomal components can be solubilized by the nonionic detergent Triton X-100 [51]. We found that the desmosomal constituents Desmoglein 1/2 and Plakoglobin displayed enhanced Triton X-100-solubility in skin from *K14CreER;Perp^fl/fl^* mice compared to skin from control mice (Figure 3B), confirming that acute deletion of *Perp* leads to impaired desmosome function similar to that observed in constitutive *Perp−/−* mice [43].

To determine whether *Perp* ablation might facilitate tumorigenesis by promoting complete desmosome loss, tumors from *Perp*-deficient mice were analyzed for the expression of Desmoglein 1/3 and Plakoglobin. Both the percentage of epithelial cells expressing each marker at the plasma membrane and the intensity of staining were measured. Staining for each antigen in tumors was categorized as high (>70%), medium (30–70%), or low (<30%) level expression. Analysis of the tumors in the *K14CreER;Perp^fl/fl^* mice revealed that the majority of lesions expressed low levels of desmosome components, suggesting that desmosome dissolution had occurred (Figure 3C, 3D). Thus, complete desmosome destabilization can occur during tumorigenesis.

To assess whether this loss of desmosomal component expression reflected a general change in differentiation status of the cells during tumorigenesis, such as Epithelial to Mesenchymal Transition (EMT) [52], we stained tumors for markers of adherens junctions. Interestingly, most cells in the tumors displayed robust membrane staining for both E-cadherin and Beta-catenin, suggesting that adherens junctions remained intact (Figure 3C, 3D). The observation that adherens junctions are maintained suggests that desmosome loss does not promote tumorigenesis through a general trans-differentiation mechanism, but rather through a more specific mechanism related to changes caused by complete desmosome-deficiency. Accordingly, staining of tumors arising in the *Perp*-deficient mice for Smooth Muscle Actin and Keratin 8 revealed a lack of expression of these markers (data not shown). Together, these data indicate that Perp loss can facilitate desmosome downregulation and that direct loss of desmosomes contributes to tumor development, but in a manner distinct from that of adherens junction dysfunction.

We also sought to establish whether desmosome destabilization occurred during SCC development in control animals by staining tumors from these mice for desmosomal proteins. Whereas non-lesional skin in the control mice exhibited normal expression of Perp, Desmoglein 1/3, and Plakoglobin (data not shown), all of the tumors from these mice displayed low level expression of one or more desmosomal components (Figure 3E). Interestingly, in this case, Desmoglein 1/3 appeared to be lost most commonly. Since desmosomes were intact at the beginning of the study, these data further suggest that targeted downregulation of the desmosome is an active characteristic of tumor development, and that weakened desmosomal adhesion, as in the case of Perp-deficiency, facilitates this downmodulation. In addition, analysis of adherens junction components in control tumors revealed intact E-cadherin and Beta-catenin expression at the plasma membrane, similar to tumors in the *K14CreER;Perp^fl/fl^* mice (Figure 3E). Thus, downregulation of desmosomal constituents with maintenance of adherens junctions is a general feature of SCC development.

### Perp expression is downregulated in human SCCs

**Figure 4:**
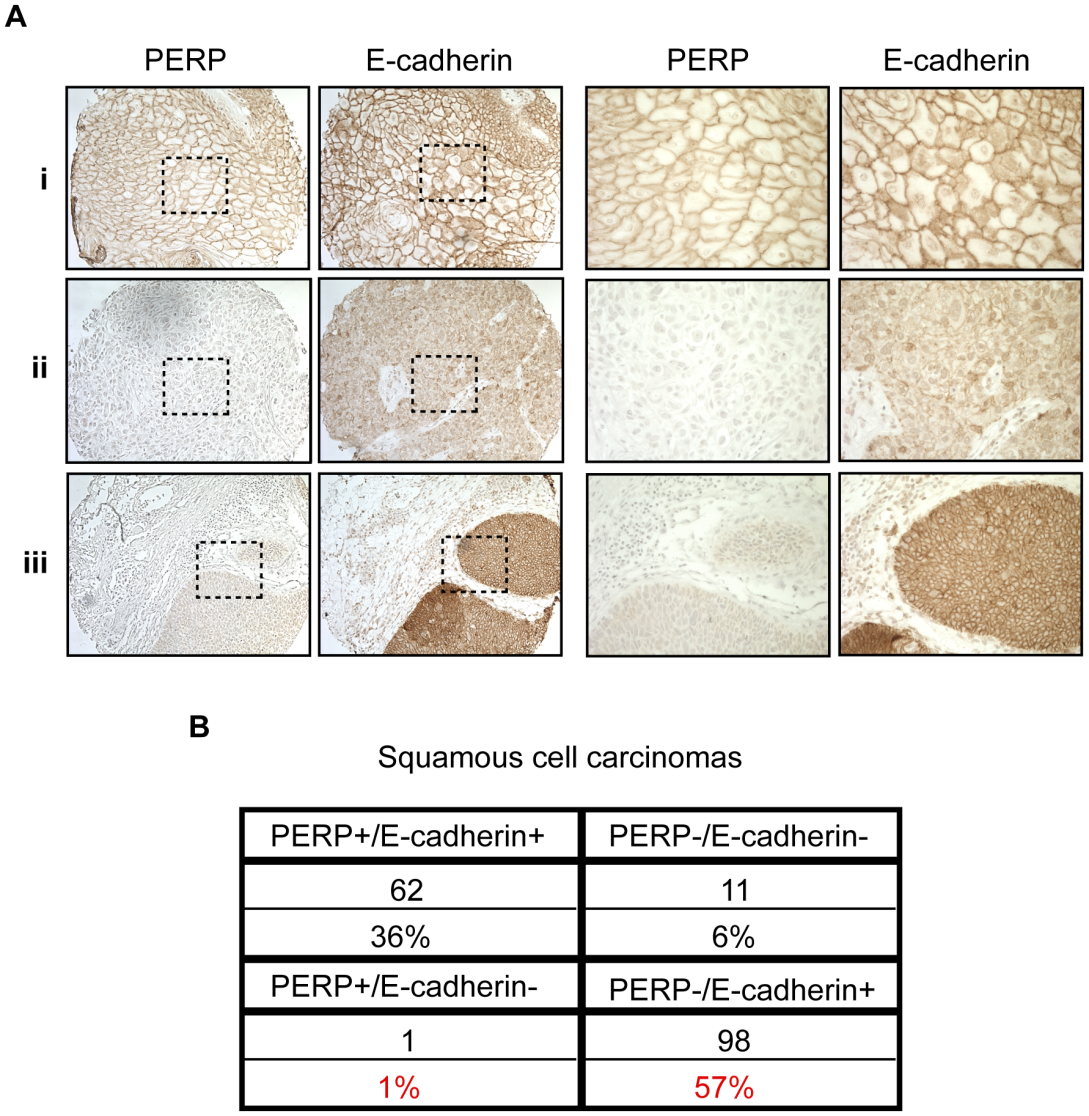
PERP loss with E-cadherin maintenance is a common event in human skin SCCs. A) Representative PERP and E-cadherin immunostaining of SCCs illustrating different expression patterns at low (left 100∶1) and high (right 400∶1) power magnification. Examples of PERP+;E- cadherin+ (i), PERP−;E-cadherin− (ii), and PERP−;E- cadherin+ (iii) tumors are shown. Dashed boxes indicate regions shown in the high magnification images. B) Table quantifying the numbers and percentages of SCCs with specific staining patterns for PERP and E- cadherin expression. Note the high percentage of tumors exhibiting strong E-cadherin staining, but no PERP staining.

Our findings suggest that desmosome loss, coupled with maintenance of adherens junctions, may represent an important stage of tumorigenesis. To determine the relevance of this observation to human SCC development, we stained a panel of samples reflecting different stages of human skin SCC development, ranging from actinic keratoses to moderately differentiated SCCs, for both PERP and E-cadherin. Each sample was scored based on the percentage of epithelial cells exhibiting membrane staining in addition to the intensity of the membrane staining in the epithelial portion of each tumor. Samples with greater than 10% of the epithelial cells expressing intense membrane staining were given a positive score, while those with less than 10% of the epithelial cells expressing either PERP or E-cadherin were given a negative score. We first noted a significant decline in the percentage of samples displaying Perp expression in the transition between AKs and SCCIS, suggesting that Perp expression is downregulated during tumor progression (p = 0.049, z-test). Interestingly, while a subset of all tumors displayed intact PERP and E-cadherin expression and another group showed complete lack of expression of both PERP and E-cadherin, the majority of tumors (57%) lacked PERP expression while retaining E-cadherin expression, akin to our observations in the mouse SCC model (Figure 4A, 4B). These findings suggest that PERP loss with retention of E-cadherin represents a significant phase of human tumorigenesis. Moreover, since the vast majority of all SCCs examined (93%) retain E-cadherin expression, and since E-cadherin loss is thought to be a late event in tumorigenesis, our data suggest a temporal sequence whereby PERP loss occurs before E-cadherin loss in the progression of human SCC.

### Perp ablation induces an inflammatory gene signature

**Figure 5:**
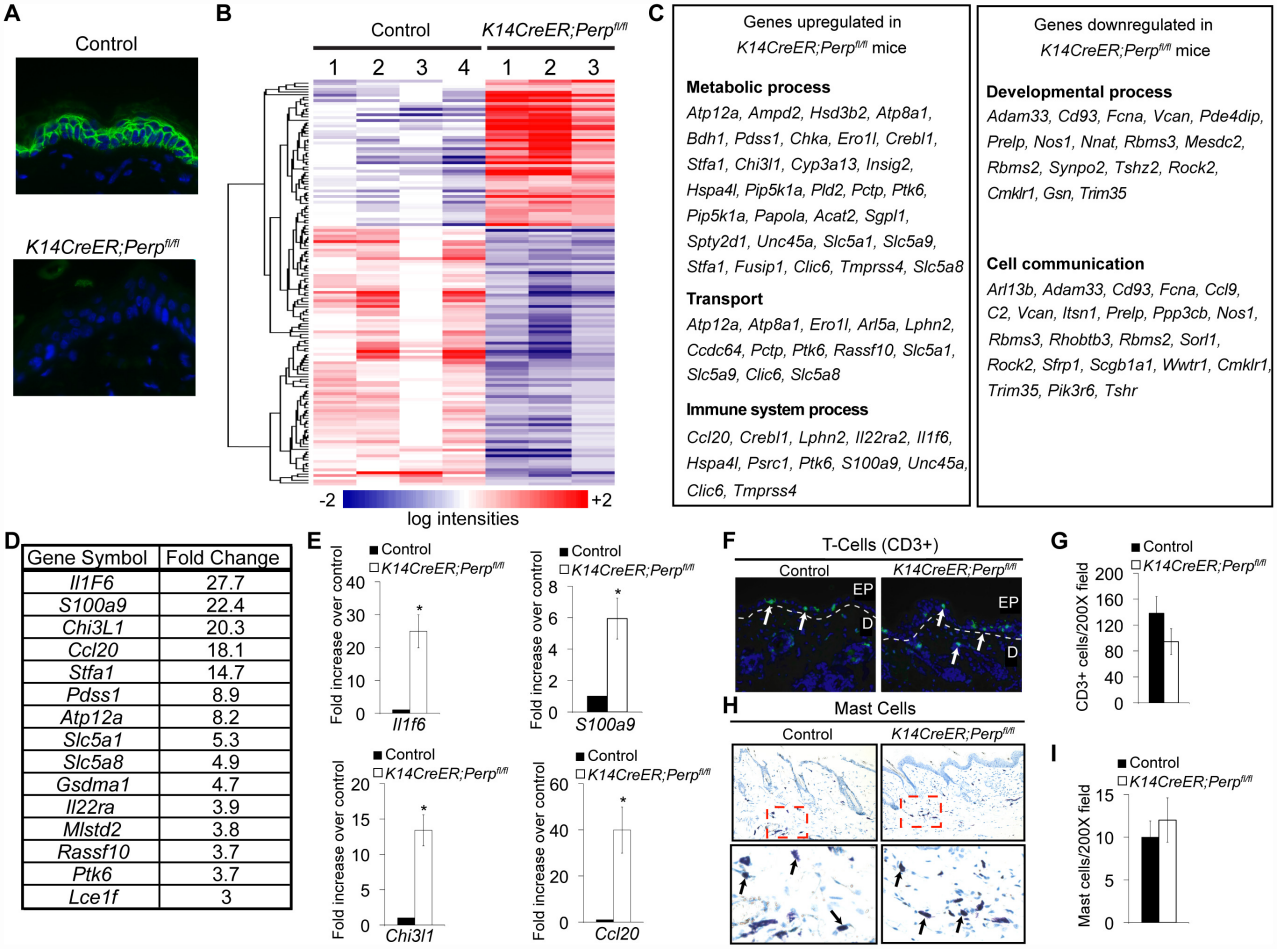
*Perp*-deficiency induces expression of inflammation-related genes. A) Perp immunofluorescence demonstrating *Perp* loss in the epidermis of *K14CreER;Perp^fl/fl^* mice two weeks after tamoxifen injection. B) Genes differentially expressed between control *K14CreER*;wild-type and *K14CreER;Perp^fl/fl^* skin were identified using SAM (Significance Analysis of Microarrays) with an FDR of 10%. The 143 genes (51 induced and 92 repressed in *Perp*-deficient skin compared to control skin) are grouped by hierarchical clustering and represented in the heat map. C) Major classes of genes upregulated and downregulated in *K14CreER;Perp^fl/fl^* skin compared to controls, as determined by Gene Ontology (GO) annotation. Members of the metabolic process, transport, immune system process, developmental process, and cell communication categories were statistically significantly enriched (p =  6.73×10^−4^, p =  3.52×10^−3^, p =  8.91×10^−3^; p =  1.59×10^−2^, p =  2.47×10^−2^, respectively, by the binomial statistic). D) Table of genes induced 3 fold or greater in *K14CreER;Perp^fl/fl^* skin relative to control samples. E) Quantitative-RT-PCR analysis validating *Il1f6* (* p = 3.1×10^−5^), *s100a9* (* p = 0.00022), *Chi3l1* (* p =  1.2×10^−6^), and *Ccl20* (* p = 0.0002) as genes induced upon Perp loss. Graphs represent the average expression levels in the skin of five mice examined in triplicate +/− SEM. Statistical significance was calculated using the Student's unpaired t-test. F) Representative immunofluorescence images of CD3-positive T-cells in control versus *K14CreER;Perp^fl/fl^* mouse skin. T-cells are stained in green (arrows) and nuclei are stained with DAPI in blue. EP indicates epidermis and D indicates dermis, with white dashed line delineating the boundary between the two compartments. G) Quantification of CD3-positive T-cells in the skin of control and *K14CreER;Perp^fl/fl^* mice. Graph represents the average number of CD3-positive T-cells counted in triplicate 200× fields, from the skin of each of at least 5 mice, +/− SEM. Statistical significance was analyzed using the Student's unpaired t-test. (p = 0.21). H) Representative images of staining for toluidine blue-positive mast cells in control and *K14CreER;Perp^fl/fl^* mouse skin. Dashed box represents area seen in higher magnification (400∶1) images below. Note that mast cells are identified by the purple stain (arrows), which differs from the blue stained background due to pH differences within mast cells. I) Quantification of mast cell numbers in the skin of control and *K14CreER;Perp^fl/fl^* mice. Graph represents the average number of mast cells counted in triplicate 200× fields, from the skin of each of 5 mice, +/− SEM. (p = 0.7; Student's unpaired t-test).

To understand how *Perp*-deficiency might cooperate with chronic UVB exposure to promote cancer, we performed microarray analyses to identify those genes whose expression is altered upon *Perp* loss. Cohorts of *K14CreER;Perp^fl/fl^* and control mice were generated and skin was processed for RNA analysis two weeks post tamoxifen injection, a timepoint at which we could first detect Perp protein expression loss throughout the epidermis (Figure 5A). Using Significance Analysis of Microarrays (SAM) [53], we identified a panel of 143 genes that were differentially regulated in the *K14CreER;Perp^fl/fl^* mice compared to controls (FDR = 10%; Figure 5B). These included 51 upregulated and 92 downregulated genes. We next classified the genes changing with altered *Perp* status based on Gene Ontology functional annotation. The three largest statistically significantly enriched groups of genes upregulated upon *Perp* ablation were in the categories of metabolic process, transport, and immune system process (Figure 5C). Significantly enriched classes of genes downregulated upon *Perp* loss included those linked to developmental processes and cell communication (Figure 5C). To identify genes whose induction might promote cancer, we examined those genes that were most highly upregulated in the absence of *Perp*. Interestingly, upon examination of the list of genes induced 3-fold or greater in the absence of Perp, we discovered that the most highly induced were several inflammation-related genes (Figure 5D). These included: Interleukin 1 Family member 6 (Il1f6), an inflammatory cytokine sufficient to induce an inflammatory response when expressed in keratinocytes of transgenic mice [54]; S100a9, a calcium binding protein with cytokine-like function in inflammation and cancer [55], [56]; Chitinase 3-like 1 (Chi3l1), a mammalian chitinase involved in enhancing inflammation as well as angiogenesis and extracellular matrix remodeling, thereby promoting tumorigenesis [57]; and Chemokine ligand 20 (Ccl20), an established chemoattractant for subsets of lymphocytes and dendritic cells which is also known to promote tumor growth [58]–[61]. Additionally, this list included Il22ra, a class II cytokine receptor and mediator of innate immune responses [62]. Quantitative RT-PCR analysis verified that the expression of these genes is indeed significantly induced in the skin of *Perp*-deficient mice (Figure 5E, data not shown).

### Chronic UVB exposure combined with Perp loss leads to the recruitment of immune cells to the skin

The induced inflammatory gene signature in Perp-deficient mice could reflect gene expression changes intrinsic to keratinocytes, or, alternatively, the recruitment of inflammatory cells to the skin of Perp-deficient mice. To distinguish these possibilities, we analyzed untreated *K14CreER;Perp^fl/fl^* and control skin samples for the presence of inflammatory cells. Histological staining for T-cells, mast cells, and myeloid cells indicated no difference in immune cell numbers between the Perp-ablated samples and the controls (Figure 5F–5I, data not shown), suggesting that Perp-deficiency induces an inflammatory gene expression program directly in keratinocytes rather than causing recruitment of immune cells to the skin.

**Figure 6:**
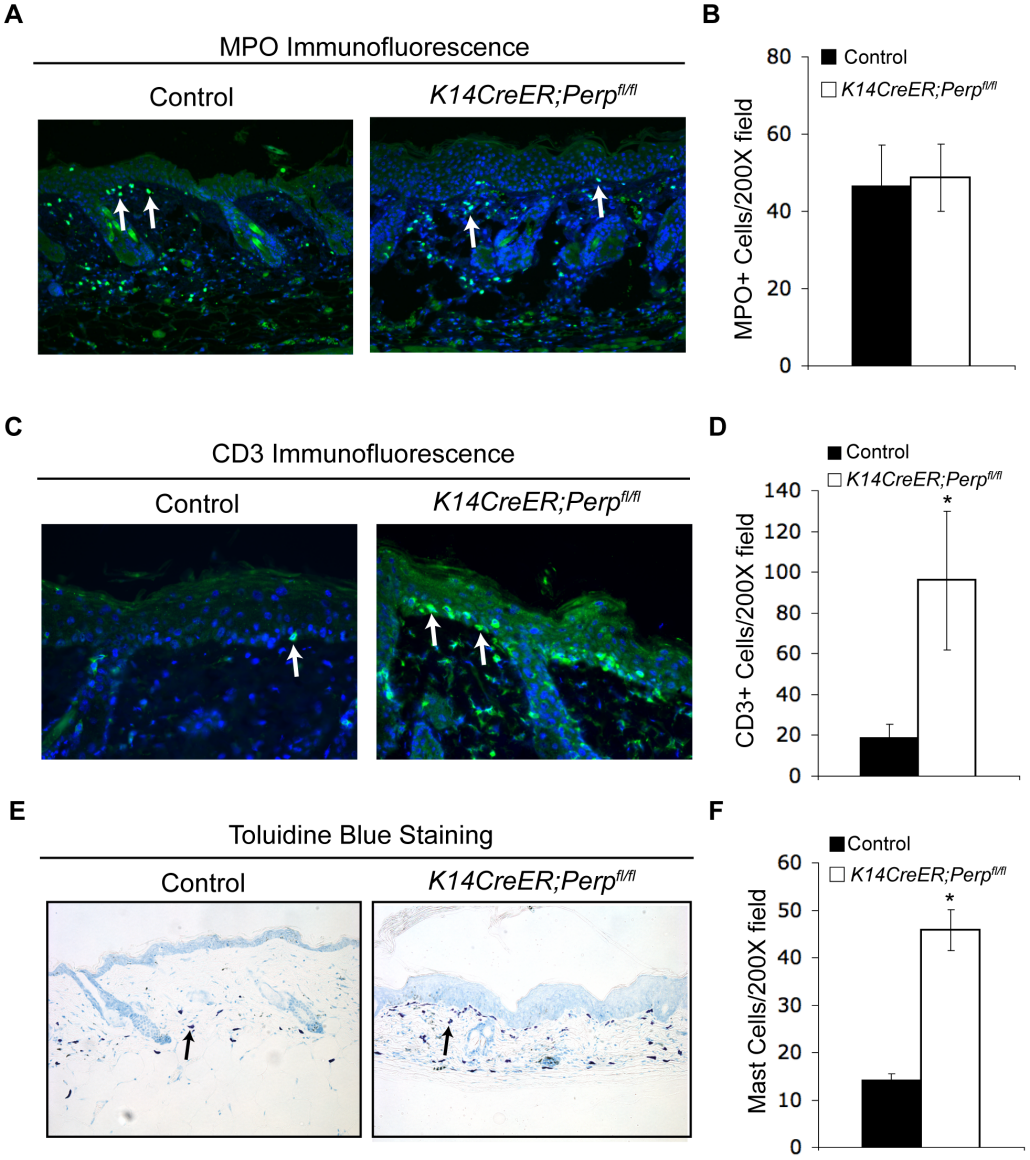
Combined Perp-deficiency and chronic UVB exposure induce immune cell infiltration in the skin. A) Representative immunofluorescence images of myeloid cells, as determined by MPO staining (arrows), in the skin of control and *K14CreER;Perp^fl/fl^* mice treated with UVB light for 19 weeks. B) Quantification of MPO-positive cells in UVB-treated cohorts. Graph represents the average of 3 mice, quantified in triplicate 200× fields +/− SEM. (p = 0.89; Student's unpaired t-test). C) Representative immunofluorescence images of T-cells, as assessed by CD3 staining (arrows), in the skin of control and *K14CreER;Perp^fl/fl^* mice treated with UVB light for 19 weeks. D) Quantification of CD3-positive T-cell numbers in UVB-treated cohorts. Graph represents the average number of T-cells in the skin of 3 mice, quantified in triplicate 200× fields, +/− SEM. (* p =  0.044, Student's unpaired t-test). E) Representative images of staining for mast cells, as assessed by toluidine blue-positivity (arrows), in the skin of control and *K14CreER;Perp^fl/fl^* mice treated with UVB light for 19 weeks. Note the increase in mast cells underlying the epidermis in the *K14CreER;Perp^fl/fl^* mice. F) Quantification of mast cells in UVB-treated cohorts. Graph represents the average of 3 mice, quantified in triplicate 200× fields +/− SEM. (* p =  0.0092; Student's unpaired t-test).

The observation that *Perp* loss triggers the induction of genes known to promote inflammation, combined with the fact that inflammation is causally linked to cancer development [63], provides a rationale for how *Perp*-deficiency could contribute to cancer. We hypothesized that persistent cytokine/chemokine signaling in *K14CreER;Perp^fl/fl^* mice, combined with chronic UVB exposure, might ultimately attract immune cells, thereby promoting tumor formation. To investigate this idea, we queried the presence of inflammatory cells in the skin from a subset of control and *K14CreER;Perp^fl/fl^* mice at an intermediate timepoint in the tumor study, after 19 weeks of chronic UVB treatment, by quantifying numbers of myeloid cells, T-cells, and mast cells (Figure 6A–6F). While we did not detect any differences in the number of myeloid cells, assessed by MPO-positivity (Figure 6A, 6B; [64]), we did observe increased numbers of T-cells present throughout the skin of *K14CreER;Perp^fl/fl^* mice compared to controls (Figure 6C, 6D). Moreover, we noted a striking increase in mast cell numbers in the skin from *K14CreER;Perp^fl/fl^* mice relative to controls (Figure 6E, 6F). As mast cells have been reported to surround tumors in a variety of cancers, including SCCs [65]–[68], and because they have been shown to play a key role in promoting tumorigenesis through the secretion of factors that remodel the tumor microenvironment and stimulate angiogenesis [68]–[71], their presence in the UVB-treated Perp-deficient mouse skin suggests an additional mechanism through which Perp loss may stimulate tumorigenesis.

## Discussion

**Figure 7:**
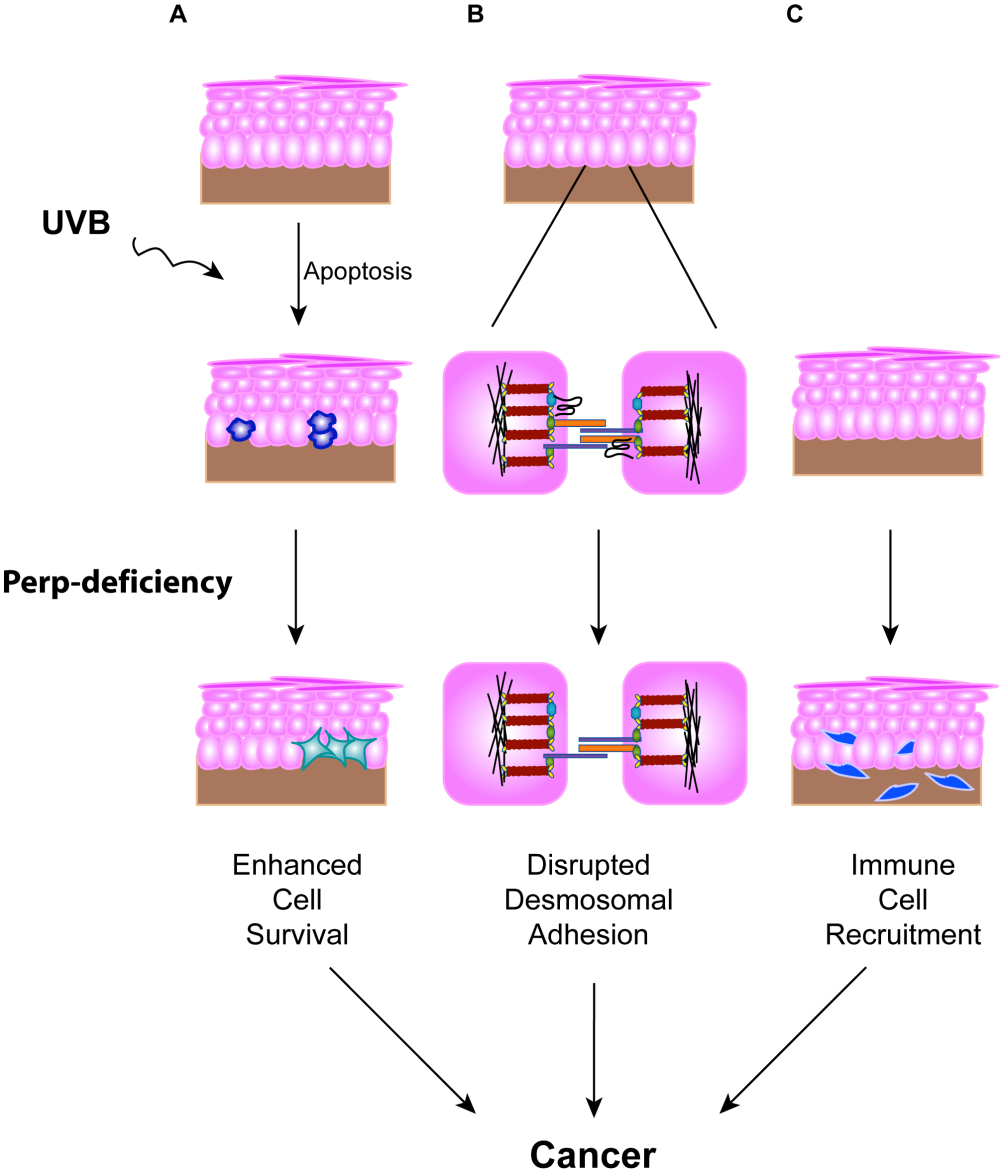
Model for how Perp-deficiency can promote tumorigenesis. Perp loss, combined with chronic UVB exposure, can promote cancer through three mechanisms. A) Compromised apoptosis in the epidermis of Perp-deficient mice in response to UVB light can lead to inappropriate survival of cells sustaining DNA damage and expansion of pre-malignant cells. B) Impaired desmosomal adhesion in Perp-deficient mice, depicted by downregulation of a desmosomal cadherin, can facilitate the complete disruption of desmosomes that stimulates tumorigenesis. The exact placement of Perp, a tetraspan membrane protein, within the desmosome is speculative. C) The recruitment of inflammatory cells to the skin of UVB-treated Perp-deficient mice can promote cancer through mechanisms such as enhancing remodeling of the tumor microenvironment or stimulating angiogenesis.

The importance of disrupted cell-cell adhesion for cancer development is underscored by the observed downregulation of adherens junction components during human cancer progression and genetic experiments demonstrating tumor prone phenotypes of mice deficient for E-cadherin, Alpha-catenin, or p120-catenin, components of the adherens junction [19]–[21]. In contrast, the data regarding desmosome protein expression during human cancer progression are conflicting [35]–[41], and the contribution of desmosome dysfunction to cancer development has not been clearly established using *in vivo* mouse models. Here, we show that loss of the desmosomal component Perp predisposes mice to UVB-induced SCC development by enhancing both tumor initiation and progression. The effects of *Perp* ablation are at multiple levels, leading both to compromised apoptosis in response to ultraviolet light and loss of desmosomal adhesion (Figure 7). The defective apoptosis could allow the inappropriate survival of damaged cells, which could help initiate tumors. The exact mechanism through which Perp promotes apoptosis remains to be elucidated, but it may relate to Perp function at the desmosome, as apoptotic defects were previously reported in cells lacking either the desmosomal component desmoglein 1 or desmoglein 2 [72], [73]. In addition, compromised UVB-induced apoptosis in the epidermis has also been observed in mice lacking the p53 target gene *Noxa*, a member of the Bcl-2 family [74]. Our findings indicate that Noxa is insufficient to drive apoptosis in the absence of Perp, and therefore that Perp and Noxa may collaborate to cause apoptosis. In addition, we observe perturbations in desmosomal adhesion. Interestingly, the desmosome downregulation we observe in tumors occurs without adherens junction loss or other signs of EMT, highlighting a specific role for desmosome loss in tumor development. It may be that desmosome loss occurs during early stages of tumorigenesis, facilitating early cancer progression, and that adherens junctions are lost subsequently, thereby promoting invasion and metastasis phenotypes. Our analysis of human SCC samples supports the idea that PERP-deficient, E-cadherin positive samples reflect an important stage of human skin carcinogenesis.

To understand further how *Perp*-deficiency might enhance tumor development, we examined gene expression profiles upon *Perp* loss. Interestingly, several of the genes induced upon *Perp* inactivation are known to be involved in promoting inflammation and tumorigenesis [54]–[62]. Inflammation is a well-established causative factor in tumorigenesis [63], as evidenced by tumor-prone mouse strains deficient for specific subsets of immune or inflammatory cells exhibiting reduced tumor burdens [66], [75]. The induction of a set of inflammation-associated genes presents a plausible explanation for how *Perp*-deficiency can promote cancer in cooperation with chronic UVB damage. Indeed, we found that Perp-deficiency, in conjunction with chronic UVB exposure, led to the infiltration of T-cells and mast cells. Mast cells can clearly promote tumorigenesis [69]–[71], and their accumulation in the UVB-treated, Perp-deficient epidermis provides another mechanism through which Perp loss can stimulate cancer development (Figure 7).

Some of the previous controversy about whether desmosomal components promote or inhibit cancer may relate to differences in the contribution of desmosomes in different contexts. Depending on the tissue type, the genetic lesions already accrued, and the tissue microenvironment, desmosome-deficiency may have different effects. Indeed, certain proteins, such as Tgf-ß1 and E2f1, can have either pro- or anti-tumorigenic roles depending on the exact setting [76], [77]. Consistent with this notion, skin carcinogenesis experiments in which *Perp*-deficient mice were treated with DMBA/TPA showed that *Perp* loss actually hindered the development of papillomas, suggesting that Perp enables the formation of this type of tumor [78]. Importantly, however, loss of the p53 tumor suppressor in this model also reduces papilloma formation, suggesting that this may represent an atypical route to tumorigenesis [79]. Therefore, in the study described here, we sought to analyze Perp function in an accurate model for human cancer, by treating mice with UVB, the causative factor for SCC of the skin.

Our studies also provide insight into mechanisms of p53-mediated tumor suppression in skin cancer. The relevance of p53 in skin cancer development is highlighted by the observations that *p53* is mutated in at least 90% of human SCCs [80] and that *p53* null mice display an enhanced predisposition to UVB-triggered skin cancer [44], [81]. While p53's ability to drive apoptosis in response to ultraviolet light has been shown to limit SCC formation [82], the molecular pathways underlying p53's tumor suppressor properties are incompletely understood. p53 is a transcriptional activator, but the genes mediating p53 tumor suppressor function have been unclear, as none of the mouse strains deficient for p53 target genes exhibits a spontaneous tumor predisposition [83]. Instead, analysis of target genes in specific contexts may reveal key functions as mediators of p53 tumor suppressor function. This notion is exemplified by studies of the apoptotic target gene *Puma*, which is important for p53 tumor suppression in the setting of Eμ-myc driven B-cell lymphomas [84]. Our studies have provided important novel insight into pathways of p53 tumor suppression by suggesting that Perp is a critical mediator of p53 tumor suppressor function in UVB-induced SCC development. In addition, although the role of p63 in cancer has been more controversial, Perp loss could also potentially explain how tumors might arise in the absence of p63.

Non-melanoma skin cancer is one of the most common malignancies in the US [85]. Fortunately, identifying SCC lesions before they progress into poorly differentiated tumors is aided both by facile detection and increased awareness of the consequences of chronic sun exposure. However, this is not the case for other stratified epithelia-derived cancers such as head and neck or esophageal cancers, which have poor survival rates [86]. Our findings may provide a framework to better understand how these more deadly diseases progress. Indeed, the loss of desmosomal component expression with maintenance of adherens junction expression observed in the *K14CreER;Perp^fl/fl^* mouse tumors and in human skin SCCs was recapitulated in samples derived from humans with head and neck SCCs (data not shown). The idea that desmosome loss may precede adherens junction loss could have important clinical implications. While E-cadherin status can provide a useful prognostic indicator for a variety of epithelial cancers, loss of this marker is associated with late-stage tumor progression [16], [87], [88]. Identifying markers like PERP that are altered earlier during tumorigenesis could potentially enhance diagnosis, grading, and prognostication, leading to more informed choices of therapy. The increased frequency of advanced tumors in the Perp-deficient mice relative to controls supports the idea that human tumors lacking PERP may ultimately progress more aggressively, and thus that PERP status may provide a useful diagnostic or prognostic marker. Indeed, previously published expression profiling studies suggest that PERP may be one in a group of key predictors for patient treatment response in esophageal cancers [89]. Further investigation of the potential diagnostic and prognostic value of PERP expression in human cancers represents an exciting avenue to pursue.

## Materials and Methods

### Ethics statement

All animal studies were approved by the Stanford University Administrative Panel on Laboratory Animal Care and were performed in strict accordance with IACUC guidelines.

### Tumor study

*Keratin-14CreER^T2^* mice were bred to *Perp^fl/fl^* conditional mice and kept on a 129/Sv; C57BL/6 mixed background [46]. At 6 weeks of age, 0.1 mg of tamoxifen (Sigma Chemical Corp., St. Louis, MO) diluted first in ethanol then in corn oil was administered to mice for 5 consecutive days via intraperitoneal injection. Four weeks post-injection, mice began chronic UVB treatments (2.5 kJ/m^2^, three times a week, for 30 weeks). Mice were shaved on a weekly basis and treated using Kodacel-filtered FS40 sunlamps. Mice were placed 5 in a cage and allowed to roam freely during treatment. Cages were rotated along the shelf below the light bulbs before each treatment to compensate for uneven distribution of energy along the bulbs. Mice were monitored for tumor incidence by visual inspection.

### Immunofluorescence/immunohistochemistry

Tissue samples were fixed overnight in 10% formalin, processed, and embedded using standard procedures. Samples were deparaffinized, rehydrated, and unmasked using Trilogy (Cell Marque, Rocklin, CA) in a pressure cooker for 15 minutes according to the manufacturer's instructions. Samples were then rinsed in phosphate buffered saline (PBS) and blocked in PBS containing 5% normal goat serum (Sigma Chemical Corp.), 2.5% bovine serum albumin (Sigma Chemical Corp.), and 0.01% Triton X-100 (Fisher Scientific, Pittsburgh, PA). Sections were incubated in primary antibody overnight at 4°C, rinsed in PBS with 0.01% Tween-20 (Fisher Scientific), incubated with secondary antibody and 1 µg/mL 4′,6-diamidino-2-phenylindole (DAPI) (Sigma Chemical Corp.) for 1 hr at 37°C, and washed in PBS. Samples were mounted with Mowiol (EMD Chemicab, Gibbstown, NJ). Fluorescence images were examined using a Leica DM6000B microscope (Leica Microsystems, Bannockburn, IL), and images were acquired using a Retiga Exi Camera (Q imaging, Surrey, British Columbia, Canada) and Image Pro 6.2 software from Media Cybernetics (Silver Spring, MD).

### Keratinocyte culture and apoptosis assays

Keratinocytes were derived from P.05–P1.5 mouse skin as described [43]. Cells were grown on collagen/fibronectin-coated dishes and maintained in an undifferentiated state by growing the cells in low calcium EMEM (Lonza, Basel, Switzerland) containing 0.05 mM calcium, 8% dialyzed FCS, and antibiotics. Cells were then differentiated for 24 hrs in the same media as the undifferentiated cells, except the calcium concentration was raised to 2 mM. For immunofluorescence, keratinocytes were grown on collagen/fibronectin-coated glass coverslips. For UVB treatment, media was removed and cells were washed once in PBS, then treated with 1 kJ/m^2^ UVB radiation using a Kodacel filter (Eastman Kodak, Rochester, NY) to block residual UVC rays. After 48 hours, cells were fixed in cold methanol for 20 minutes at −20°C. Cells were stained with rabbit anti-cleaved Caspase 3 antibodies (Cell Signaling, Beverly, MA), followed by staining with FITC-anti-rabbit antibodies (Vector Laboratories, Burlingame, CA) and DAPI and mounting using Mowiol.

### *In vivo* apoptosis assays

Cohorts of *K14CreER;Perp^fl/fl^* mice were generated, and at 6 weeks of age mice were injected with tamoxifen as described above. Four weeks later the dorsal skin of mice was shaved. Mice were placed underneath a Kodacel filter and allowed to roam freely in their cage during UVB treatment. Half of the dorsal skin was exposed to a one time dose of 2.5 kJ/m^2^ of UVB irradiation while the other half was blocked. 24 hours later the dorsal skin of the mice was collected and immunostained for cleaved Caspase 3. Apoptosis, indicated by cleaved Caspase 3-positivity, was quantified in at least 2–3 cm of skin per mouse.

### Antibodies

Primary antibodies against Perp [43], Desmoglein 1 (Santa Cruz Biotechnology, Santa Cruz, CA), Plakoglobin (clone 11E4; Invitrogen), Keratin 14 (Covance, Princeton, NJ), Alpha-tubulin (Sigma Chemical Corp.), E-cadherin (Invitrogen), GAPDH (Fitzgerald Industries, Acton, MA), Plakoglobin (1408; gift of K. Green), Smooth Muscle Actin (Santa Cruz Biotechnology), Desmoglein 1/3 (clone 32-2B; gift of D. Garrod), Desmoglein 1/2 (4B2; gift of K. Green), cleaved Caspase 3 (Cell Signaling), p53 (Vector Laboratories), Beta-catenin (BD Biosciences, San Jose, CA), Desmoplakin (11-5F; gift of D. Garrod), CD3 (Dako, Denmark), and MPO (AbCam, Cambridge, MA) were used in this study. Secondary antibodies used were: FITC-anti-mouse and FITC-anti-rabbit (Vector Laboratories) and TRITC-anti-chicken, HRP-anti-mouse, and HRP-anti-rabbit (Jackson ImmunoResearch, West Grove, PA).

### Solubility assays

For the solubility assay, skin samples were frozen and ground up, resuspended in a 0.1% Triton X-100-based solution, and nutated for 1 hr at 4°C [43]. The insoluble pellet was lysed in 9M urea buffer. Samples were then analyzed using conventional western blotting protocols.

### Human tissue microarrays

Archival paraffin embedded tissue blocks were retrieved from the Dermatopathology Section of the Department of Pathology and Dermatology, University of California, San Francisco and from outside pathology laboratories. Tumor-bearing regions from paraffin-embedded, formalin-fixed tissue samples were identified by a dermatopathologist using routine hematoxylin and eosin stained sections. Tissue microarrays comprising 0.6 mm cores of skin actinic keratoses, skin carcinomas *in situ*, skin SCCs, and adjacent normal tissue samples were constructed. Sections (5 µm) were cut and placed onto Superfrost plus slides (Fisher Scientific) [90]. 23 AKs, 20 SCCIS, and 160 SCCs were analyzed.

### Microarray analysis

RNA was isolated from the dorsal skin of control and *K14CreER;Perp^fl/fl^* mice two weeks post-tamoxifen injection using Trizol (Invitrogen), according to the manufacturer's protocol. RNA samples were processed at the Stanford University Pan Facility and analyzed using Affymetrix Mouse Genome 430 2.0 expression arrays. Probe-level data were processed using BRB-ArrayTools (Biometric Research Branch of the National Cancer Institute) based on RMA (Robust Multichip Average) for background adjustment, normalization and expression summarization. Class comparison analysis was conducted using SAM (Significance Analysis of Microarrays) with an FDR (False Discovery Rate) of 10% [53]. Gene function categorization based on GO (Gene Ontology) was carried out using the PANTHER (Protein ANalysis THrough Evolutionary Relationships) classification system.

### Quantitative reverse transcription PCR

Skin from control and *K14CreER;Perp^fl/fl^* mice was isolated two weeks after tamoxifen injections. RNA was isolated using Trizol (Invitrogen), and 1 µg RNA was reverse transcribed using Moloney Murine Leukemia Virus reverse transcriptase (Invitrogen) and random primers. PCR was performed in triplicate using SYBR green (SA-Biosciences, Frederick, MA) and a 7900HT Fast Real-Time PCR machine (Applied Biosystems, Foster City, CA), and results were computed relative to a standard curve made with cDNA pooled from all samples. Values were first normalized to β-actin and then represented relative to untreated wild-type samples. Five mice per genotype were analyzed. Primers used were as follows: *β-actin* 5′-TCC TAG CAC CAT GAA GAT CAA GAT C-3′, Rev 5′-CTG CTT GCT GAT CCA CAT CTG-3′, *S100a9* 5′-GGA AGG AAG GAC ACC CTG AC-3′, Rev 5′-CCA GGT CCT CCA TGA TGT C-3′, *Il1f6* 5′-CTG TTC TGC ACA AAG GAT GG-3′, Rev 5′-GCT GCA GAC TCA AAT GTA GAG G-3′, *Il22ra* 5′-GGA CAC CCC GCT TCA CTC-3′, Rev 5′-ATT TGG CAA CTC TGG AGG AC-3′, *Chi3l1* 5′-AGC AGT ATT TCT CCA CCC TGA T-3′, Rev 5′-CGC TGA GCA GGA GTT TCT CT-3′, and *Ccl20* 5′-AAC TGG GTG AAA AGG GCT GT-3′, Rev 5′-GTC CAA TTC CAT CCC AAA AA-3′.
